# Clinical and radiological results of cruciate-retaining total knee arthroplasty with the NexGen®-CR system: comparison of patellar resurfacing versus retention with more than 14 years of follow-up

**DOI:** 10.1186/s13018-017-0646-4

**Published:** 2017-10-02

**Authors:** Keun Churl Chun, Sung Hyun Lee, Jong Seok Baik, Seng Hwan Kook, Joung Kyue Han, Churl Hong Chun

**Affiliations:** 1 Department of Orthopedic Surgery, School of Medicine, Wonkwang University, 895, Muwang-Ro, Iksan, 54538 South Korea; 2 Collage of Sports Science, Chung-Ang University, Anseong, South Korea

**Keywords:** Total knee arthroplasty, Cruciate retaining, Patellar resurfacing, Patellar retention

## Abstract

**Background:**

The purpose of this study is to analyze clinical and radiological outcomes of patients (with a minimum of 14 years of follow-up) who underwent cruciate-retaining (CR) total knee arthroplasty (TKA) using a NexGen®-CR, comparing a patellar resurfacing group with a patellar retention group.

**Methods:**

From June 1996 to April 2002, 116 cases of TKA using a NexGen®-CR who had at least 14 years of follow-up were enrolled in this study. Among them, 68 cases had patellar resurfacing and 48 had patellar retention. The average follow-up period was 14.8 years (14.1–18.7). Clinical scores and range of motion (ROM) were evaluated preoperatively and at the last follow-up in all patients. The Hospital for Special Surgery (HSS) score, Knee Society Score (KSS), Western Ontario and MacMaster Universities Osteoarthritis (WOMAC) score, and a new patellar score were assessed. Radiological evaluations are done by analyzing the tibiofemoral angle, loosening, and a radiolucent line on the radiograph by American Knee Society Roentgen Graphic Evaluation.

**Results:**

The average HSS score of both the patellar resurfacing group and retention group increased from 42.3 and 41.2 preoperatively to 90.2 and 90.8 at the last follow-up, respectively. The KSS, WOMAC score, patellar score, and knee joint ROM also improved significantly in both groups. However, there were no significant differences in clinical results between the two groups. On the radiological evaluation, the tibiofemoral angle in both groups had improved from varus 7.8° and 7.2° preoperative to valgus 4.9° and 4.8°, respectively. The average angles of *α*, *β*, *γ*, and *δ* were 94.1°, 90.4°, 3.2°, and 87.8° in the patellar resurfacing group and 94.4°, 89.8°, 3.3°, and 88.1° in the patellar retention group, respectively. A radiolucent line shown on radiograph was noted in a total of seven cases, three in the patellar resurfacing group and four in the patellar retention group. In the patellar resurfacing group, among the seven zones on the tibia radiograph, all cases were located at the medial side of tibia and two cases were in zone 1 and one case in zone 2, and in the patellar retention group, three cases were in zone 1 and 1 case was in zone 2, also located on the same side.

**Conclusions:**

We achieved satisfactory clinical and radiological outcomes on long-term follow-up when performing TKAs with a NexGen®-CR. There was no significant difference in clinical or radiological results between the patellar resurfacing and retention groups in our study.

## Background

Total knee arthroplasty (TKA) is widely performed in order to reduce the pain that is caused by a joint destruction due to joint problems that are accompanied by functional disorders and, in addition, to promote a stable joint movement by correcting deformities [1]. With the advancements that have been made in surgical techniques and equipment design, the long-term survival rate of the prosthetic joint has increased when compared to that when the procedure was first introduced [1, 2].

Since Freeman et al. [3] introduced the procedure of posterior cruciate ligament (PCL) resection in 1977, the decision whether to retain the ligament during a TKA procedure has been a point of controversy from various perspectives. If the posterior cruciate ligament is retained during a TKA operation, the flexion range of motion (ROM) of the knee can increase because a femoral rollback occurs during the flexion like a normal knee. The posterior cruciate ligament is the strongest ligament in the knee, and therefore, if it is retained during a TKA operation, the knee’s original stability can be preserved post procedure [4]. Additional advantages of a posterior cruciate ligament retention are that patients are more functional when walking and climbing stairs because of a better proprioception [1]. The loosening of the implant is also less likely because the ligament reduces the friction between the implant and the bone [4, 5].

The patella in the patellofemoral joint bears up to seven times the body weight during joint exercises. Hence, when considering the various functions and symptoms involving the patella, whether to retain it during a TKA surgical process remains controversial [6]. Waters and Bentley [7] argued that patellar resurfacing had a better outcome in terms of pain reduction, with a patient’s satisfaction and less complications, whereas Burnett et al. [8] argued for the retention of the patellar, and Jung et al. [9] advocated for selective patellar resurfacing.

Therefore, in the current study, we aimed at comparing the clinical and the radiological outcomes of patellar resurfacing against patellar retention by evaluating the long-term follow-up results over at least 14 years of post cruciate retention when using the NexGen®-CR system for a TKA operation.

## Methods

### Study subjects

We analyzed the clinical and the radiological outcomes of 116 out of 124 patients who underwent a cruciate-retaining TKA operation when using a NexGen®-CR system, which was not consisted of patella friendly femoral prosthesis, from June 1996 to April 2002. They were followed up for at least 14 years. Eight patients were excluded due to death (3 cases) and 5 cases were lost to follow-up. Of the patients that were included in the study, 40 patients were men and 76 patients were women. The mean length of the follow-up was 14 years and 8 months (14.1–18.7 years). Patellar resurfacing was performed in 68 cases, while patellar retention was performed in 48 cases (Table 1).

**Table 1:** Demographics of total patients and patients with patellar resurfacing and patellar retention TKAs

	Patellar resurfacing	Patellar retention	*p* value
Number of patients	68	48	
Age (years)	64.2 (54–81)	63.8 (54–72)	0.218
Sex (male to female)	7:61	2:26	
Follow-up (years)	14.2 (14.1–18.7)	15.2 (14.3–18.5)	
Body mass index (kg/m^3^)	26.1 (21–36)	26.3 (22–35)	0.367

Values are presented as mean (range), *p* value < 0.05

*TKA* total knee arthroplasty

### Surgical approach and rehabilitation

A single surgeon performed the procedure for all of the patients in the study. Medial parapatellar skin incision and arthrotomy was used. A measured resection technique was used in order to perform the osteotomy on the proximal tibia and the distal femur. An extramedullary alignment guide was used for the tibia, whereas an intramedullary alignment guide was used for the femur. An effort was made in order to obtain a tibial posterior slope angle of 3°–5°, and the distal femur was resected at 7° valgus. All of the posterior femoral osteophytes were removed in order to prevent the femoral implant from impinging on them when the knee was excessively flexed. A posterior cruciate ligament release was performed at the site of the femoral attachment if the implant was likely to be lifted off because of a small flexion gap. In all of the cases, cement was used for a fixation. The reports from Vanlommel et al. [10], Vaninbroukx et al. [11], and other researchers have indicated that it is favorable to apply cement with a depth to the cancellous bone and to a length of the cement mantle. So in the cases when using cement on an implant and a bone, a full cement technique was used in order to apply the cement onto the femur, the tibia, the patella, and the implant.

**Fig. 1:**
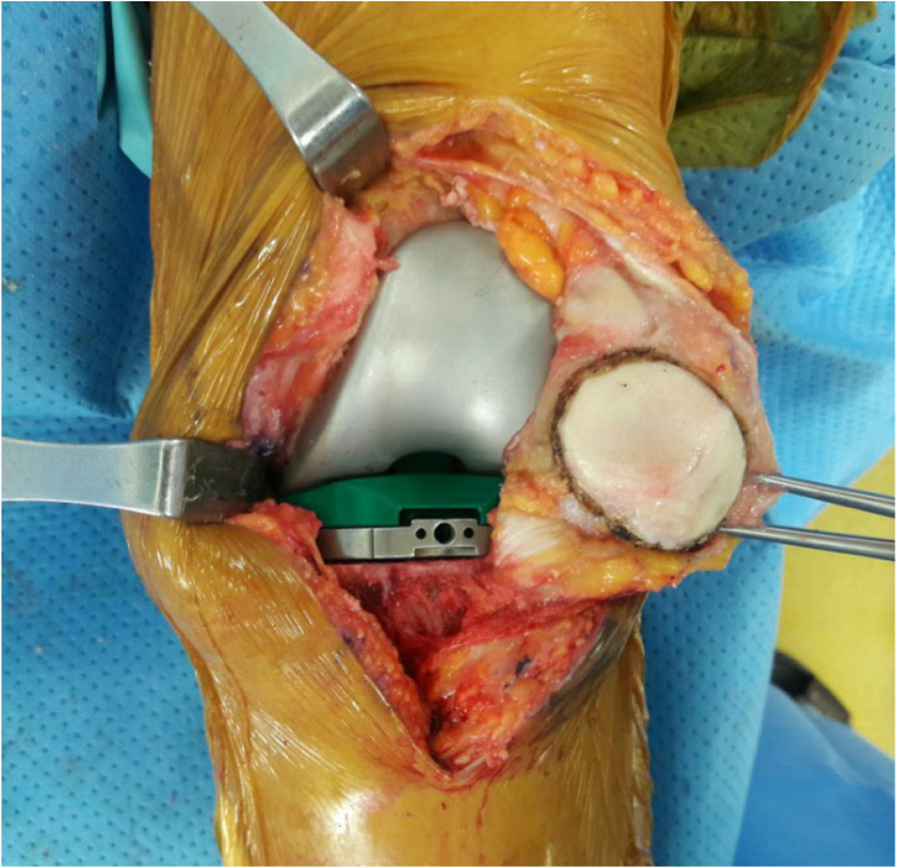
In patellar retention group, the osteophyte removal and the chondroplasty, as well as a synovectomy on the thickened synovium surrounding the patella, and coagulation for denervation were all performed

Whether or not to resurface the patella was determined according to the level of pain in the anterior patellar area prior to the operation and the extent of the cartilage damage that was confirmed during the procedure. We performed patella resurfacing in patients who had more than grade 3 of arthritic changes in trochlear, medial facet, or proximal half of patella [12, 13]. When performing patellar resurfacing, the patella was resurfaced using a standard cemented polyethylene patellar button. When not performing a patellar resurfacing, the osteophyte removal and the chondroplasty, as well as a synovectomy on the thickened synovium surrounding the patella, were all performed. Especially, the osteophyte removal and the patelloplasty of lateral facet of patella should be done. Also, a monopolar bovie electrocautery was used on coagulation at 40 to 60 W and run around the periphery of the patella at approximately 50% of its depth for 20 to 30 s (Fig. 1).

Two days after the procedure, the drainage tube was removed and the patients began a continuous passive motion exercise and an active exercise for the knee. They started walking with a walker. Two weeks after the procedure, the patients started to walk, bearing their entire body weight without a walker. Additionally, they were recommended to carry out more everyday activities when standing up, by using furniture for stability, and to use the knee within the limits of its natural ROM.

### Clinical and radiological evaluations

For the clinical evaluation, the patella scores and the functional scores were obtained both before the operation and at the final follow-up examination. The evaluations were conducted regarding pain, functionality, and the ROM. The angle of the deformed joint was assessed, by using the scores that included the Hospital for Special Surgery (HSS) scores, the Knee Society Score (KSS) scores, the Western Ontario and MacMaster Universities Osteoarthritis (WOMAC) scores, and the Kujala patella scores [14].

**Fig. 2:**
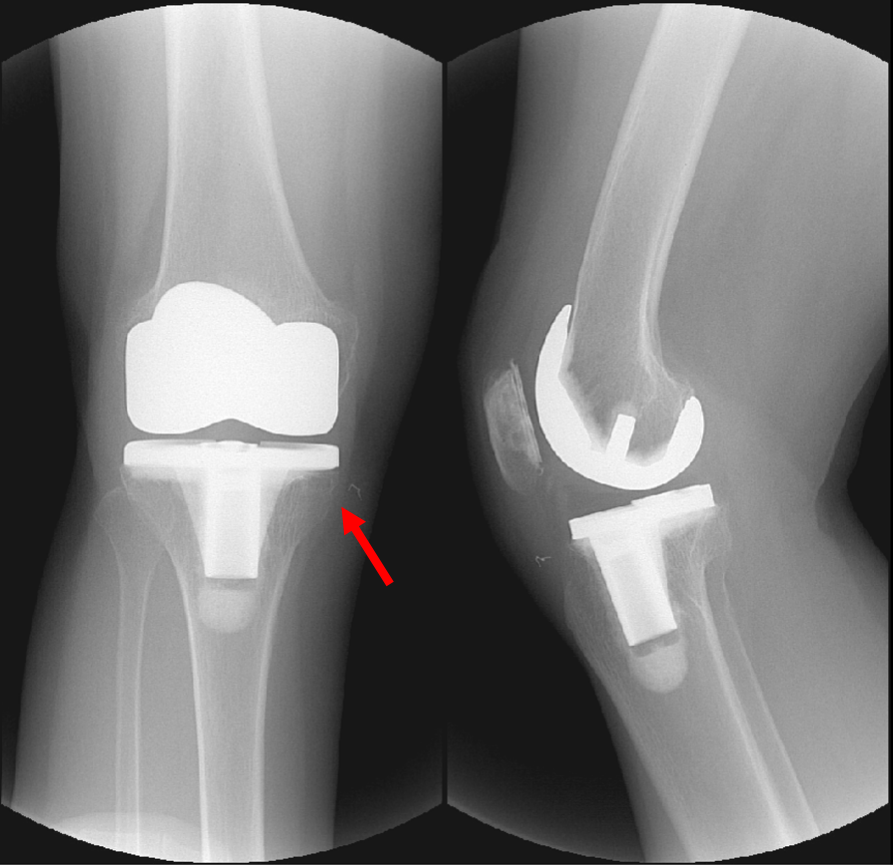
The radiolucent line and loosening were evaluated by fluoroscopy. The radiolucent line is shown at the medial side of the tibia (zone 1)

For the radiological evaluation, the femoral-tibial angle, the loosening of the implant, and the radiolucent lines were all analyzed by using the American Knee Society Roentgenographic Evaluation method [15]. At preoperative, postoperative, and final follow-up, anteroposterior and lateral images of the upright standing posture were taken. Anteroposterior and lateral fluoroscopy were both performed in order to examine the radiolucent lines (Fig. 2). The presence or the absence of a radiolucent line that was wider than 2 mm was identified, and any collapse or dislocation of the implant was measured. The implant location was determined by measuring the femoral-tibial angle, the valgus angle of the femoral implant (α), the varus angle of the tibial implant (β) on an anteroposterior viewing of the knee, the flexion angle of the femoral implant (γ), and the posterior slope angle of the tibial implant (δ) on a lateral viewing of the knee [15].

In order to decide on the presence of radiolucent lines, the contact surface between the bone and the cement or between the bone and the prosthetic joint implant on the radiographic images was at first divided in the following manner: seven zones in the lateral surface of the femoral implant, seven zones in the anteroposterior surface, three zones in the lateral surface of the tibial implant, and five zones in the lateral surface of the patellar implant. The presence of radiolucent lines was defined by a radiolucent line over 2 mm wide that appeared in any of the zones. Implant loosening was defined by categories, including a case where the radiolucent lines gradually increased in width, a case in which the radiolucent lines appeared, and a case in which the implant was dislocated [15].

### Statistical analysis

The preoperative and postoperative patellar scores were compared by using the paired *t* test. The ROM scores and the scores for the patellar joint of both of the groups, with and without a patellar resurfacing, were compared by using Student’s *t* test. All of the statistical analyzes were performed with SPSS Software (Version 12.0, SPSS, Chicago, IL), and the statistical significance was defined as *p* < 0.05. When power analysis was performed to evaluate the power of group comparison for clinical scores and ROM, this study achieved a power of 0.82 for detection of differences with actual *α* = 0.05. G*Power 3.1.9.2 was used for the power analysis.

## Results

### Clinical outcomes

Clinical scores improved significantly in both cohorts following TKA surgery, though there were no significant differences seen between the two cohorts. The HSS scores of the entire sample of patients improved from a mean of 42.0 before the operation to a mean of 90.4 at the final follow-up. The scores improved from 42.3 to 90.2 in the patellar resurfacing group (group I) and from 41.2 to 90.8 in the patellar retention group (group II). The KSS scores of the entire sample of patients improved from a mean of 39.5 to a mean of 87.8. The scores improved from 39.8 to 87.6 in group I and from 38.6 to 88.2 in group II. The arthritis symptoms improved in the entire sample of patients from a mean WOMAC score of 85.5 to a mean WOMAC score of 32.9. The scores changed from 85.8 to 32.5 in group I and from 84.7 to 33.7 in group II. The Kujala patella scores of the entire sample of patients increased from a mean score of 42.3 to a mean score of 70.6. The scores improved from 41.8 to 71.5 in group I and from 43.7 to 70.1 in group II. The ROM of the entire sample of patients was increased from a mean of 109.2° to a mean of 123.4°. Group I showed an increase from 111.8° to 123.6° and group II showed an increase from 108.2° to 122.8° (Table 2). The mean ROM scores, the patellar scores, and the functional scores all showed significant differences in the preoperative and postoperative comparisons (all were *p* < 0.05). However, groups I and II did not show significant between-group differences relating to the preoperative and postoperative ROMs, the Kujala patella scores, or the functional scores (all were *p* > 0.05).

**Table 2:** Clinical results of patients with patellar resurfacing and patellar retention TKAs

	Preoperative status			Postoperative†		
	Total	Patellar resurfacing‡	Patellar retention‡	Total	Patellar resurfacing‡	Patellar retention‡
KSS	39.5	39.8	38.6	87.8	87.6	88.2
HSS	42.0	42.3	41.2	90.4	90.2	90.8
WOMAC	85.5	85.8	84.7	32.9	32.5	33.7
Patellar score	19.5	18.7	19.9	26.1	25.6	27.8
ROM	109.2	111.8	108.2	123.4	123.6	122.8

Data are presented as means, with ranges in parentheses, or scores

*TKA* total knee arthroplasty, *KSS* Knee Society Score, *HSS* Hospital for Special Surgery, *WOMAC* Western Ontario and MacMaster Universities Osteoarthritis, *ROM* range of motion

†Clinical results of postoperative status were significantly improved compared to preoperative status (*p* < 0.05 on paired t-test)

‡No significant differences were found between patellar resurfacing and patellar retention groups (*p* > 0.05 on Student’s *t* test)

### Radiological outcomes

When viewing the preoperative radiological anteroposterior images of the full weight-bearing standing, the mean femorotibial angle was 7.6° varus for the entire sample. It was 7.8° varus in group I and 7.2° varus in group II. At the final follow-up, the mean angle was 4.9° valgus for the entire sample. It was 4.9° valgus in group I and 4.8° valgus in group II (*p* = 0.001). The implant location was estimated based upon the postoperative radiological anteroposterior images, and the mean valgus angle of the femoral implant (*α*) was 94.2°, the mean varus angle of the tibial implant (*β*) was 90.2°, the mean flexion angle of the femoral implant (*γ*) was 3.2°, and the mean posterior slope angle of the tibial implant (*δ*) was 87.9°, for the entire sample of patients. The mean *α*, *β*, *γ*, and *δ* angles in group I were 94.1°, 90.4°, 3.2°, and 87.8°, respectively. The corresponding angles in group II were 94.4°, 89.8°, 3.3°, and 88.1°, respectively. The location and the alignments of the implants were well maintained. A comparison of the radiographical images that were taken both immediately after the procedure and at the final follow-up revealed a change of less than 5°.

All of the patients underwent a fluoroscopy at the final follow-up in order to check for radiolucent lines in each of the aforementioned zones, and the locations of the lines were recorded. A distribution of radiolucent lines was found on the anteroposterior and lateral view in seven cases, with three cases in group I and four cases in group II. One case in group I had radiolucent line in zone 1 out of the seven zones on lateral surface of the femur, one case had radiolucent lines in zone 1 on the anteroposterior surface of the tibia, and one case had radiolucent lines in zone 2 on the same surface. In group II, all four cases also had radiolucent lines in the lateral side of their tibia, with three cases in zone 1 and one case in zone 2 on the anteroposterior surface of their tibia (Table 3). There were no cases that showed a significant loosening of the implant, such as a collapse or a dislocation and functional problems. Finally, there were no significant differences in any of the comparisons that were tested between the group I and group II (all were *p* > 0.05).

**Table 3:** Distribution and incidence of radiolucent lines in the femoral and tibial components

Radiology	Zone (patellar resurfacing/patellar nonresurfacing)							Incidence
	1	2	3	4	5	6	7	
Femur lateral	0/0	0/0	0/0	0/0	0/0	0/0	0/0	0
Tibia AP	2/3	1/1	0/0	0/0	0/0	0/0	0/0	3/4
Tibia lateral	0/0	0/0	0/0	0/0	0/0	0/0	0/0	0

Data are presented as number of cases

### Complications

**Fig. 3:**
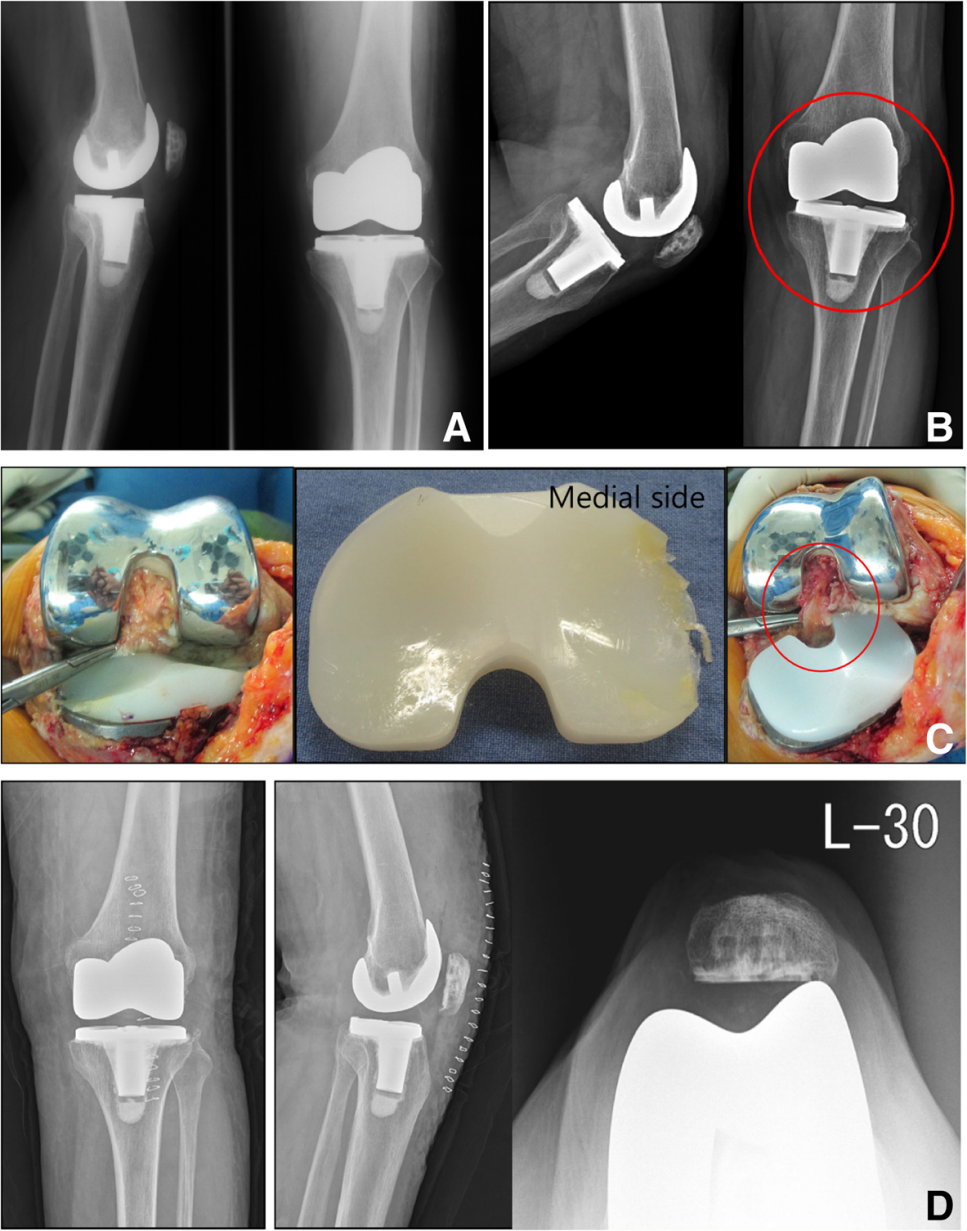
**a** A 63-year-old woman with osteoarthritis. Postoperative radiograph. **b** Radiograph taken 15 years after surgery shows wear of meniscus bearing. **c** Intraoperative finding shows wear of meniscus bearing and an intact posterior cruciate ligament, so isolated meniscus bearing was diagnosed. **d** Radiograph taken after revision surgery

**Fig. 4:**
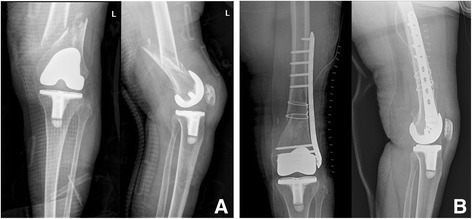
At 16 years after total knee arthroplasty (TKA), the patient visited our hospital for knee pain after a fall. **a** The radiograph shows a periprosthetic fracture around the prosthesis. **b** Postoperative radiograph taken after open reduction and internal fixation with a plate and wiring

In group I, three types of complication occurred—polyethylene wear (one case), a deep infection (one case), a periprosthetic fracture (one case), and patella subluxation (three cases). In group II, two cases developed a deep infection. A two-stage revision arthroplasty was performed in the two cases that had a deep infection, whereas a polyethylene replacement was only done in the one case with polyethylene wear. This was because the posterior cruciate ligament was preserved without any loosening of the implant, the alignment of the lower limbs was normal, and the structures of the soft tissue were balanced (Fig. 3). The one case with a periprosthetic fracture underwent an open reduction and an internal fixation procedure (Fig. 4). The three cases with patella subluxation did not undergo procedures such as replacement since there was no anterior knee pain or functional problem.

## Discussion

The goals of a TKA procedure are focused on pain relief, stabilization of the joint, and correcting any deformities. With the recent advancements in scientific tools for prosthetic knee joints and surgical techniques, the 15- to 20-year long-term survival rate of the prosthesis is as high as 90–98% in both young and elderly patients [16].

The NexGen®-CR (Zimmer, Warsaw, Indiana, USA) system, a prosthetic knee joint that was developed in the mid 1990s, has an incurvate shape, with a small radius that assures a wide contact with and a good conformity to the femur condyle. Additional characteristics of the system are the combination of a diverse array of implants; an increment of the contact area and conformity to the joint by an anatomically designed femoral implant with improved knee tracking; compatibility between the different sizes of implants; and the selection of a variety of surgical instruments in order to perform the procedure. In addition, a change can be made from PCL retention to a PCL substitution, according to the need during the procedure [17].

Surgeons who prefer to retain the posterior cruciate ligament have argued that the ligament is the strongest part in the knee and that posterior tibial subluxation can be prevented by retaining it in order to preserve the original stability. In addition, they argue that proprioception is better with posterior cruciate ligament retention, and therefore, there is a better function when walking and climbing stairs [18]. The balance in the muscles around the knee is also better through an appropriate perceptual feedback [19]. In contrast, a posterior cruciate ligament resection has been reported to have the advantages of an easier surgical operation, a minimization of the tibial resection margin, an avoidance of tension in the ligament, a wide contact with the joint in order to reduce the likelihood of polyethylene wear, and an easier deformity correction [20, 21].

Lowry and Sledge [22] have argued that a posterior cruciate ligament resection is preferred for those patients with a varus deformity of more than 25°, or a knee flexion contracture of more than 30°, if the ligament is deformed. Accordingly, in the present study, we have used these conditions as the surgical criteria in determining whether to retain or resect the posterior cruciate ligament. So far, there is no clinical index available in order to determine the condition of the posterior cruciate ligament. Hence, we have tried to minimize the errors in a case selection, by having a single surgeon making consistent decisions across all of the cases with regard to the function of the posterior cruciate ligament and on the basis of a visual inspection and a tension test. In our clinical practice, we have experienced satisfying outcomes with posterior cruciate ligament retention. Because of the lack of a histological index to help a surgeon determine the condition of the posterior cruciate ligament and the difficulty in making such a decision, we decided that a visual inspection with a test for the tension of the ligament could serve as a clinical index when determining whether to retain the ligament in a TKA procedure.

In the current study, we found that the ROM significantly improved from a mean of 109.2° to a mean of 123.4° post procedure in those patients who underwent a cruciate-retaining TKA that was performed with the NexGen® system and with a prosthetic knee joint. The prosthetic survival rate after a mean follow-up period of 14 years and 8 months was 95.7%. These outcomes are satisfactory when in comparison with those of other prosthetic knee replacement surgeries.

The importance of an axial alignment between the femur and the tibia after a TKA operation has been stressed in the literature. For example, Lotke and Ecker [23] have shown that a position of between 3° and 7° valgus was desirable. We observed a satisfactory level of deformity correction from the preoperative alignment of 7.6° varus to the postoperative alignment of 4.9° valgus.

Kraay et al. [24] reported that radiolucent lines were most frequently seen in the most proximal area of an anterior flange. They speculated that this might be due to nonconformity of the femoral implant to the anterior resection surface of the femur and the insertion of a flexed implant. King and Scott [25] reported that 15 out of approximately 1600 cases of a TKA procedure experienced a loosening of the femoral implant. An implant loosening occurred in zone 4 in 13 cases, and radiolucent lines were observed immediately after the procedure in 8 cases.

They argued that the early loosening of the femoral implants that were fixated with cement was caused by a lack of support in the posterior condyle area of the femoral implant. This was because of an incomplete resection of the posterior femoral condyle, a poor cementing technique, and an insufficient structure of the condyle. However, when analyzing the influence of radiolucent lines on patients, we observed radiolucent lines in zones 1 and 2 (the lateral side of the tibia on the anteroposterior surface), finding different results than the study of King and Scott. We believe that this was because we used a full cement technique to apply the cement onto the femur, the tibia, the patella, and the implant. No cases in our study showed aseptic loosening.

Patellar resurfacing is a subject of much controversy [4, 26]. However, it has been reported that patellar resurfacing is preferred in those cases who suffer from severe patellofemoral arthritis, rheumatoid arthritis, a poor patellofemoral alignment, a patellofemoral pain prior to surgery, and an abnormal alignment and height of the patella. Shih et al. [27] reported that they observed degenerative changes in the patellofemoral joint and with a valgus displacement in those cases with a preoperative patellar maltracking. They argued on the basis of their findings that patella maltracking could be an indication for a patellar resurfacing. However, they did not find an association between the radiological findings and the patellar scores. Other researchers have also reported a lack of significant differences in the clinical outcomes between patellar resurfacing and patellar retention [1, 19]. In the present study, we did not find significant differences, clinically or radiologically, between the patellar resurfacing group (68 cases) and the patellar retention group (48 cases).

In patella resurfacing group, there were three (4.4%) patients who showed patellar subluxation. There were several reasons which influenced on patellar subluxation after patellar resurfacing surgery. One of the reasons was the consequent tension of the lateral side. Resection of the lateral facet or the distal pole leads to tightness of the lateral retinaculum and a tendency to subluxation [28, 29]. However, there was no symptomatic subluxation which led to anterior knee pain or functional problem. Therefore, we did not perform an additional surgical procedure. Conservative methods as quadriceps exercises, braces, or avoiding activities that aggravate instability were applied in subluxations and with time scarring of the retinacular tissues lead to resolutions of the symptoms.

There are a few limitations in the present study. First, it was retrospectively conducted with only one type of implant. Second, the decisions on performing either patellar resurfacing or patellar retention were made by the surgeon’s subjective judgment. However, this study is of significance in that it reported the long-term follow-up results regarding the NexGen®-CR system. This particular procedure was used for posterior cruciate ligament retention. In addition, the clinical outcomes of patellar resurfacing and patellar retention were compared in all of the patients when using the same implant system.

## Conclusion

The survival rate of the implants after a TKA operation when using the cruciate-retaining NexGen®-CR system after a long-term follow-up of more than 14 years was good. There were no significant differences in the clinical or the radiological outcomes between the patellar resurfacing and the patellar retention.
